# Potential Phenotyping Methodologies to Assess Inter- and Intravarietal Variability and to Select Grapevine Genotypes Tolerant to Abiotic Stress

**DOI:** 10.3389/fpls.2021.718202

**Published:** 2021-10-26

**Authors:** Luísa C. Carvalho, Elsa F. Gonçalves, Jorge Marques da Silva, J. Miguel Costa

**Affiliations:** ^1^LEAF – Linking Landscape, Environment, Agriculture and Food – Research Center, Associated Laboratory TERRA, Instituto Superior de Agronomia, Universidade de Lisboa, Lisboa, Portugal; ^2^BioISI – Biosystems and Integrative Sciences Institute, Faculty of Sciences, Universidade de Lisboa, Lisboa, Portugal

**Keywords:** heat and water stress, imaging, phenotyping planning, planting material, selection traits, *Vitis vinifera*

## Abstract

Plant phenotyping is an emerging science that combines multiple methodologies and protocols to measure plant traits (e.g., growth, morphology, architecture, function, and composition) at multiple scales of organization. Manual phenotyping remains as a major bottleneck to the advance of plant and crop breeding. Such constraint fostered the development of high throughput plant phenotyping (HTPP), which is largely based on imaging approaches and automatized data retrieval and processing. Field phenotyping still poses major challenges and the progress of HTPP for field conditions can be relevant to support selection and breeding of grapevine. The aim of this review is to discuss potential and current methods to improve field phenotyping of grapevine to support characterization of inter- and intravarietal diversity. *Vitis vinifera* has a large genetic diversity that needs characterization, and the availability of methods to support selection of plant material (polyclonal or clonal) able to withstand abiotic stress is paramount. Besides being time consuming, complex and expensive, field experiments are also affected by heterogeneous and uncontrolled climate and soil conditions, mostly due to the large areas of the trials and to the high number of traits to be observed in a number of individuals ranging from hundreds to thousands. Therefore, adequate field experimental design and data gathering methodologies are crucial to obtain reliable data. Some of the major challenges posed to grapevine selection programs for tolerance to water and heat stress are described herein. Useful traits for selection and related field phenotyping methodologies are described and their adequacy for large scale screening is discussed.

## Introduction

The EU is the leading global wine producer, with about 44% of the world’s vine-growing area (*circa* 3.2 million ha) and sustaining about 57% of wine production by volume (OIV, 2020). European Mediterranean countries lead the cultivated area of grapevine for wine production worldwide (OIV, 2020) but they are also increasingly exposed to more adverse weather conditions, with air temperatures rising from 2 to 5°C in major winemaking regions in parallel with changes in precipitation patterns or/and higher frequency of extreme weather events, such as heat waves (IPCC, 2014; Fraga, 2020; Lorenzo et al., 2021).

These changes have a serious impact on the sustainability of the wine sector in Mediterranean countries (e.g., Spain, France, Italy, Greece, and Portugal). Several agronomic strategies are already being implemented in viticulture to face climate challenges, and adapt to more severe heat and drought. The use of deficit irrigation is one of the most common (see Santesteban et al., 2019 for a review), but several others have been proposed and reviewed (see Gutiérrez-Gamboa et al., 2021 or Naulleau et al., 2021), and their economic consequences for the producers were analyzed (Merloni et al., 2018).

The use of better adapted plant material is another priority, namely in terms of late ripening varieties (Wolkovich et al., 2018), heat/drought tolerant clones (Van Leeuwen et al., 2013; Bota et al., 2016), and rootstocks adapted or modified to forthcoming climate conditions (Ollat et al., 2016; Prinsi et al., 2021). However, field phenotyping and grapevine selection are laborious and expensive, and still pose major challenges. The progress of high throughput plant phenotyping (HTPP) for field conditions can be relevant to support selection and breeding of grapevine. Therefore, the aim of this review is to identify potential strategies and methods to improve field phenotyping of grapevine to support characterization of inter- and intravarietal diversity. In fact, *Vitis vinifera* has a large genetic diversity that needs characterization to support selection of better adapted plant material (polyclonal or clonal), namely to abiotic stress.

### The Impact of Heat and Water Stress on Grapevine Physiology

Stomatal behavior is a crucial functional trait and stomatal responses to the environment are determinant for plant adaptation. Stomata influence CO_2_ uptake into the leaf along with water loss due to transpiration, actively regulating plant water status and leaf temperature (Jones, 1992; Matthews and Lawson, 2019). Stomata respond to chemical stimuli (biochemical control due to hormonal control) and to leaf water status (hydraulic control; Pantin et al., 2013) that mediate environmental inputs, such as light intensity and quality, air CO_2_ concentration, and vapor pressure deficit (VPD; Buckley, 2019). Increasing soil water use is associated with hydraulic traits, to enable gas exchange under more negative water potentials, as observed by Dayer et al. (2020) in Semillon. Stomatal conductance to water vapor (*gs*) on Chardonnay did not respond to air temperature below 30°C, but dropped under a combination of high air temperature and high air VPD (Greer, 2020). Different stomatal behaviors have been described for other varieties, thus the interaction between air temperature and VPD must be considered when addressing stomatal responses (Dayer et al., 2020; Greer, 2020).

Some varieties show a tight stomatal control (isohydric), whereas others show a less efficient stomatal control in response to water stress (anisohydric). Nevertheless, such classification of *Vitis* varieties as isohydric or anisohydric remains controversial since differences in stomatal behavior among varieties are far more complex and largely depend on growing conditions (Chaves et al., 2010; Lovisolo et al., 2010; Villalobos-Gonzalez et al., 2019; Gambetta et al., 2021). In fact, it was shown that a variety can behave as both iso- and anisohydric, according to the level of water deficit, which defies the standard classification that implies a single behavior (Levin et al., 2019). Gambetta et al. (2020) suggested a more integrative definition of drought tolerance in grapevine, by resorting to four core physiological traits: maximum transpiration rate; stomatal regulation (expressed as the relation between stomatal conductance and leaf water potential); turgor loss point; and root volume. Bringing these parameters together, the authors suggested that it is possible to calculate, at any moment, for a vineyard under defined environmental conditions, the “stress distance,” i.e., the amount of time (e.g., number of days) that it withstands without watering before reaching a critical water potential.

The plasticity of leaf morphology is another factor of adaptation and evolution (Fritz et al., 2018). The role of leaf epidermis characteristics (cuticle, indumentum, pavement cells, and stomata) and mesophyll anatomy can have an impact on responses to abiotic stresses (Tomás et al., 2014; MacMIllan et al., 2021). Leaf morphology and structure may affect stomatal behavior, leaf gas exchange, and mesophyll conductance (Tomás et al., 2014). Stomatal density and stomatal index can influence varietal leaf gas exchange characteristics as well as thermal regulation capacity (Gago et al., 2019). Costa et al. (2012) found no differences in stomatal density between Cabernet Sauvignon, Touriga Nacional, Syrah, Trincadeira, and Aragonez (syn. Tempranillo), but reported differences in *gs*, leaf temperature, and leaf photosynthesis, suggesting that other factors besides the number of stomata regulate leaf gas exchange in grapevine. Gago et al. (2019) reported that Grenache Noir had significantly smaller leaf surface area than Syrah, but significantly thicker leaf blades. This calls for improved knowledge on morphological, anatomical, and physiological traits influencing the response to heat and drought of the *Vitis* germplasm.

The role of abscisic acid (ABA) in stomatal closure is well established; this hormone plays a key role particularly in isohydric or near-isohydric plants (Sampaio Filho et al., 2018; Dayer et al., 2020), by inducing faster ABA-related gene modulation (dal Santo et al., 2016). Stomatal sensitivity to ABA is variable among varieties (Rossdeutsch et al., 2016; Simonneau et al., 2017). Rossdeutsch et al. (2016) concluded that *Vitis* sp. genotypes with contrasting levels of drought adaptation differ in key steps involved in ABA metabolism and signaling, both when well-watered and drought stressed.

Grapevine’s photosynthetic apparatus is defined as resilient, but extreme climate conditions will affect it negatively, through the overreduction of the photosynthetic electron carriers, production of reactive oxygen species (ROS), and photoinhibition (Mittler, 2006). In Mediterranean summer conditions, grapevine plants growing under heat and drought are usually exposed simultaneously to photoinhibitory light conditions, high air temperatures, and moderate to severe soil water deficits (Carvalho and Amâncio, 2019). If stress persists and carbon fixation is reduced, oxidative stress may take place (Carvalho and Amâncio, 2019). When drought co-occurs with high light intensities an increase in ROS production by the photosynthetic apparatus can also arise (Mullineaux et al., 2006), leading to photoinhibition of photosynthesis.

Heat stress physiology in turn, at both leaf and berry levels, should be evaluated to better understand the impacts of drought and high soil and air temperatures on grapevine physiology and morphology of leaves, berries, and bunches (Costa et al., 2019a; Field et al., 2020). This is particularly important because berries tend to ripe earlier in warmer conditions, due to the effect of heat in anticipating phenological events (Van Leeuwen and Destrac-Irvine, 2017).

Plant phenology and growth are largely driven by air temperature and soil water availability (Parker et al., 2011). In fact, Verdugo-Vásquez et al. (2020) developed a climate-based model to estimate grapevine phenology, taking into account meteorological data and microclimate data at the plant level. Concomitantly, berry composition is affected by water availability and heat, with extreme temperatures and severe drought affecting negatively vigor, yield, and berry composition (Chaves et al., 2010), such as a lower content of anthocyanins (van Leeuwen and Darriet, 2016; Zarrouk et al., 2016). In addition, acidity, in particular related to malic acid content, decreases in high air temperature (van Leeuwen and Darriet, 2016). Consequently, the modern wine industry must find adequate varieties to maintain berry quality traits, such as acidity, under extreme and adverse climate conditions. Aspects such as berry sensitiveness to drought and sunburn were recently revised by Gambetta et al. (2021), attesting the relevance of the problem for the academy and the industry.

### The Role of Plant Material to Mitigate Stress and Decrease Risks of Combined Heat Waves and Drought

**Figure 1:**
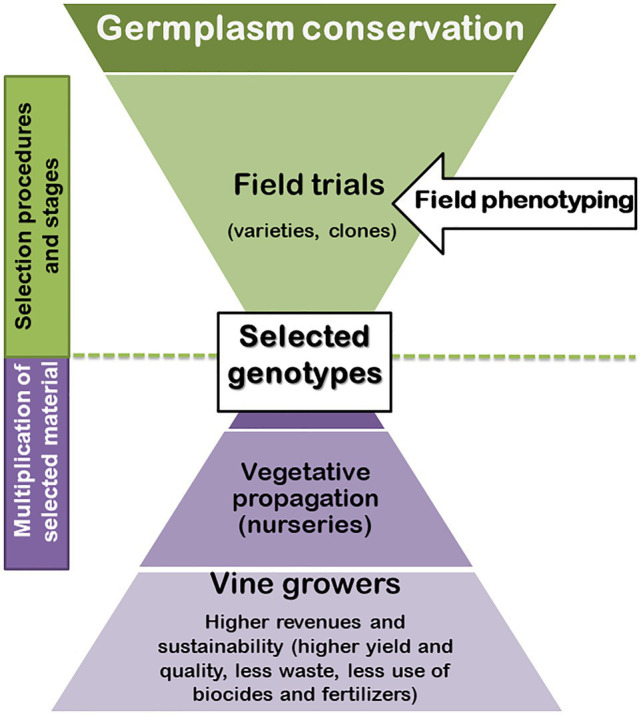
Illustration of the added value of phenotyping to the procedure of grapevine selection.

Using optimal adapted plant material (rootstocks and *V. vinifera* varieties) for a specific region is a long term adaptation strategy crucial for grower’s revenue and sustainability of the sector (less water, pesticides, and fertilizers required; Figure 1). Grapevine has a high level of phenotypic plasticity and genotypes can respond by adapting their growth morphology, leaf gas exchange, and berries’ metabolic characteristics. Such plasticity was recently reported in a three season study of 30 varieties, indicating possible adaptations to climate change, such as the earlier and shorter ripening phase of white varieties to avoid the warmest period of the season (Gashu et al., 2020).

Autochthonous grapevine varieties represent a strong natural and historical mark, add great value to top quality wines, and are an essential raw-material to face future challenges. Therefore, a better characterization of existing variability between and within varieties is necessary, especially if we consider the need to adapt to scenarios of climate change. Usually, varieties original from the Mediterranean basin are perceived as drought tolerant, such as the widely-used Grenache, Cinsault, Carignan, Cabernet Sauvignon, Sangiovese, Zinfandel, and Nebbiolo (Fraga et al., 2012; Van Leeuwen et al., 2019), and the less extensively spread Xinistery from Cyprus (Van Leeuwen et al., 2019). Some Portuguese varieties have also been described as well adapted to abiotic stress, such as Cerceal-Branco, Encruzado, Touriga Franca, and Viosinho (Carvalho et al., 2017). Furthermore, the existence of intravarietal variability in grapevine is the available resource for polyclonal selection (Resolution OIV-VITI 564B-2019; OIV, 2019) and clonal selection (OIV-VITI 564A-2017; OIV, 2017) aiming at climate change adaption.

Despite having a small land area, Portugal is extremely rich in autochthonous varieties. As a result, a coherent strategy has been developed to stop the ongoing erosion of intravarietal genetic diversity of all autochthonous varieties, to improve methods of conservation, to evaluate the intravarietal diversity for selection focused on yield, important must quality traits, and tolerance to abiotic and biotic stresses (Martins and Gonçalves, 2015; Gonçalves et al., 2016; Carvalho et al., 2020). This strategy has been implemented in the field by the National Network for Grapevine selection and by the Portuguese Association for Grapevine Diversity (PORVID).

The resources available in Portugal to perform field phenotyping to select superior clones within Portuguese grapevine varieties comprise a network of more than 185 field trials of 63 varieties, distributed along the country, and established according to efficient experimental designs to carry out selection. Rootstocks influence resistance to abiotic stress, namely to drought (Pavlousek, 2011; Harbertson and Keller, 2012). The combination of tolerant rootstocks with tolerant clones could be the most effective long term strategy to overcome adverse climate limitations that currently affect Portugal and other Southern European countries (Santos et al., 2020). The graft-scion incompatibility remains a major issue as it can limit response to heat and drought (Tedesco et al., 2020).

Fast, robust, and accurate screening of specific traits to assess tolerance to abiotic stress of rootstocks and *V. vinifera* varieties is crucial to obtain plant material able to cope with climate change. Phenotyping technologies (for controlled and field conditions) have undergone great progress in the last decade. The latest innovations and respective application to different crops have been intensively described (Araus et al., 2018; Qiu et al., 2018; Das Choudhury et al., 2019; Pieruschka and Schurr, 2019; Roitsch et al., 2019; Jiang and Li, 2020; Li et al., 2020; Moreira et al., 2020; Yang et al., 2020; Jin et al., 2021). A multiple set of methods and technologies are now available to support the evaluation of quantitative traits, including crop yield and tolerance to abiotic stresses. In this review, the available phenotyping methodologies will be analyzed in light of their potential use to evaluate inter- and intravarietal variability and to support selection of grapevine genotypes for tolerance to abiotic stress.

## Phenotyping in Grapevine

### Definitions, Scales, and Approaches

Phenotyping is the process of systematically determining, analyzing, and predicting all or part of an organism’s phenotype, and the concept was used for the first time in the 1950s. However, it was only in 2013 that Fiorani and Schurr (2013) coined the term “plant phenotyping,” defining it as “*the set of methodologies and protocols used to accurately measure plant growth, architecture, and composition at different scales*.” Phenotyping aims at providing valuable data to improve management of biodiversity resources, to foster crop/variety adaptability to the environment and resistance against pests and diseases (Costa et al., 2019b,c) as well as to identify superior traits such as yield and quality. Phenotype, as the result of the genotype (G), the environment (E), and the interaction between them (G×E) is dynamic, complex and comprises multiple quantitative traits that make it hard to study, and especially, to quantify.

Phenotyping methodologies and procedures to characterize and select individuals with particular traits and clear advantages at the level of stress resistance, yield performance, and fruit quality traits, require a systematic approach and organized data collection to facilitate further analysis. Plant phenotyping can be carried out at different levels of biological organization with similar aims but yielding different outputs. Molecular phenotyping involves transcriptomics, proteomics, metabolomics, and related areas such as lipidomics, and can be targeted to single-cell phenotyping, in which the effects of a mutation can be studied through changes in a single cell (Schiefelbein, 2015). On the other end, there are field and ecosystem level phenotyping.

Molecular phenotyping focuses on the investigation of gene function and/or biochemical pathways underpinning physiological mechanisms affecting development, productivity, and stress responses. In grapevine, it aims at developing biotechnology programs to scan for tolerance (Ciaffi et al., 2019) or to develop improved varieties that enable the production of specific wines (DeBolt et al., 2006; Harris et al., 2013) or that are tolerant to biotic stresses (Agüero et al., 2005). At this level, phenotyping approaches are often destructive and require extensive sample manipulation and processing. Therefore, the concept of HTPP refers mainly to whole-plant phenotyping and is largely based in automated image capture and analysis [e.g., Red, Green, Blue (RGB), thermal, multispectral, and fluorescence imaging].

Modern HTPP platforms are coupled to controlled environment growth facilities, allowing large scale screening, isolation of the genetic component of the phenotype, and selection of the most promising genotypes. Large scale plant phenotyping has been extensively studied and developed under controlled conditions, especially for screening of model plants such as Arabidopsis (Merlot et al., 2002), but also for cereals (Raskin and Ladyman, 1988), canola (Knoch et al., 2019), or pepper (Toledo-Martín et al., 2016). In the last few years, good progress was made in the use of remote and proximal sensing tools to meet the phenotyping needs of annual crops, namely by using automated multi-sensor phenotyping machines.^1^ Such platforms are sophisticated and costly. Therefore, low-cost or more cost-effective phenotyping options are being developed (Reynolds et al., 2019), among them, user-built cost-controlled prototypes (e.g., in the project INTERPHENO^2^).

However, as crops are subjected to multiple stresses, changing in duration and intensity along time, selected genotypes must be tested under conditions that are more realistic, namely in field conditions. Field phenotyping is complex, since environmental conditions cannot be controlled and it is difficult to homogenize sampling conditions. Also, field phenotyping infrastructures are not easily available due to their high costs. Some initiatives have been implemented to use field phenotyping technologies based on ground and aerial platforms. For example, the European project EMPHASIS, which is on its implementation phase, aims to create a permanent European HTPP infrastructure network, has a work package fully dedicated to field phenotyping.^3^

Under the plant-breeder’s perspective, an efficient phenotyping must take into account two standpoints: (1) the availability of adequate tools to measure the target traits and (2) the planning of phenotyping. Concerning the first aspect, plant breeding needs simple, fast and HTPP methods well adapted to the main agronomic, physiological, and technological traits. The second aspect is related to the actions before and after phenotyping, that is, the rules that must be followed to ensure that the obtained data can be suitable for an efficient use of the acquired measurements, namely, for selection purposes and comparative experiments. This is particularly relevant to feed biodata infrastructures (ex. EU ELIXIR project or BioData.pt. which is the Portuguese distributed infrastructure for biological data).

## Current Technologies and Strategies to Screen Grapevine Germplasm

The need to identify grapevine varieties/genotypes with specific characteristics that enable them to deal with challenges posed by climate change is universally recognized. Nevertheless, we are still far from having reliable, fast, and efficient methodologies for grapevine phenotyping at reduced cost, especially in field conditions. The use of phenotyping devices in woody perennial crops with complex canopies and architecture, such as grapevine still poses difficulties. However, the principle of using indirect non-contact measurements to quantify physiological traits is suitable for grapevine field phenotyping. This approach is getting more attention in parallel with the increasing availability of proximal and remote sensing technologies, especially for “stress-tolerance” based on imaging (RGB, thermal, chlorophyll fluorescence, and hyperspectral). There is also an increasing number of available tools for image processing and analysis, and of algorithms that can support a phenotyping decision (Tsaftaris et al., 2016; Barradas et al., 2021), some developed specifically for grapevine (de Castro et al., 2018; di Gennaro et al., 2019). The potential applications of different imaging approaches for selection and stress monitoring are briefly described below and summarized in Table 1.

**Table 1:** List of imaging methodologies, their advantages, disadvantages, and potential application to screen grapevine plants in field conditions (genotype selection for yield, berry quality, and for abiotic stress).

Imaging methods	Phenotyping traits	Organ	Advantages	Disadvantages	Potential use for selection	
					Yield/quality	Abiotic stress
RGB visible	Morphology and Growth Leaf color Necrosis	Roots Leaves Canopy Berries Clusters Shoots Trunk	Multiple solutions at low cost Fast and user friendly High portability and multiple platforms Assessment of biotic and abiotic stress Highly adequate for field measurements Easy image analysis and processing	Slightly (in structural/morphological analysis) to significantly (in color analysis) disturbed by light conditions Limited output on physiological data Only 2D Limited feasibility under field conditions for root analysis	No	Yes, but needs optimization for pre-selection
Thermal infrared	Morphology and Growth Canopy and leaf temperature Stomatal behavior Necrosis	Leaves Canopy Berries Clusters Trunk	Multiple solutions and prices High portability and multiple platforms Assessment of biotic and abiotic stress Adequate for field measurements	Impact of environment [radiation, wind speed, Tair and air vapor pressure deficit (VPD), and rain], background; Wet and dry references may induce errors Image analysis and processing (software still expensive and demanding skills)	No	Yes, but needs optimization for pre-selection
Multispectral	Leaf color Chlorophyll content Carotenoid content Secondary metabolites (anthocyanins, terpenoids) Necrosis	Leaves Canopy Berries	Assessment of biotic and abiotic stress Adequate for field Some information on biochemical traits	Expensive equipment Plant architecture and light conditions may influence analysis Low capturing speed Difficult image analysis	Yes	Yes
Hyperspectral	Leaf color Chlorophyll content Carotenoid content Secondary metabolites (anthocyanins, terpenoids) Necrosis	Leaves Canopy Berries	Assessment of biotic and abiotic stress Adequate for field Some information on biochemical traits	Highly expensive equipment Growth of the plant and illumination influence analysis Lack of exhaustive info on biochemistry tissue Difficult image analysis	Yes	Yes
Chlorophyll fluorescence	Photosynthetic efficiency Leaf senescence degree Oxidative stress Membrane integrity	Leaves Berries	Assessment of biotic and abiotic stress Adequate for field (point measurements)	Expensive equipment, with limited portability Complex to image full and deep canopies Strong impact of environment (light conditions)	No	Yes, but needs extensive optimization for pre-selection
Laser, stereo (LiDAR)	Plant biomass Plant structure Leaf area, angle, and composition	Leaves Canopy Trunk	3D images Assessment of biotic and abiotic stress Adequate for field	Expensive Demanding skills Lack of exhaustive info about plant physiology	Yes, but needs extensive optimization	No

### Visible RGB Imaging

Shoot growth, leaf area, and yield are important agronomical and morphological parameters used for different crops. Traditionally they are quantified by weighing or manually measuring shoot elongation and leaf area. Despite the ease of these measurements, they are very time-consuming and inadequate for large scale phenotyping. RGB imaging, currently widespread in consumer-grade digital cameras and mobile phones, but also available in a diversity of industrial devices tailored for artificial vison (Pajares et al., 2016), is an efficient method to assess leaf area and yield in grapevine (Mabrouk and Sinoquet, 1998; Nuske et al., 2011; Diago et al., 2012; Arnó et al., 2013; Dogan et al., 2018).

Pipeline image analysis performed after automatic selection of representative pixels for each category, such as “soil,” “leaves,” “wood,” or “grapes,” showed high correlation, for leaf area and fruit yield, with the values obtained by destructive methods (Diago et al., 2012). RGB images also proved to be a feasible tool to estimate yield. Berry detection was based not only on the color but also on berries geometry, specifically the radial symmetry, to distinguish them from the background even when green (Nuske et al., 2011; Abdelghafour et al., 2019; Briglia et al., 2019). More recently and using a robot as movable ground platform, Victorino et al. (2020) collected image-based indicators to support yield prediction at different phenological stages in grapevine. The authors reported that bunch volume and bunch projected area had significantly high correlation coefficients with yield, regardless of the fruit’s occlusion.

Red, Green, Blue images have also been used to estimate the whole plant leaf area (LA) and fresh biomass in grapevine (Coupel-Ledru et al., 2014), both relevant traits to assess plant vigor. Regarding fruits, cluster compactness is an important trait to select table and wine grapes and the assessing methodology is based on the OIV descriptors, using morphological features of the clusters, and can also be estimated using image analysis, a faster and non-destructive alternative of characterization (Palacios et al., 2019). These authors developed a mobile sensing platform that automatically captures RGB images of grapevine canopies and fruiting zones at night using artificial illumination.

The distinction between the individual plants in the foreground and the vineyard in the background poses a major challenge for sensor-based phenotyping, particularly when RGB images are used, since similar color distributions occur in both. To overcome this difficulty, Klodt et al. (2015) developed a method of background subtraction based on taking two images of each plant for depth reconstruction, which were then successfully used to evaluate 3D leaf surface areas and the ratio fruit-to-leaf in new grapevine breeding lines.

Faster image data retrieval will be crucial to gain efficiency in field phenotyping. Imaging acquisition using more or less complex ground-based platforms (robots, tractors, and quads) must still be optimized for the vineyard. This poses major challenges namely related to irregular and rocky soils, different plant spacing, and orientation. Due to the frequency of image acquisition and the storage capacity, driving speed for data acquisition in field conditions has been limited to 0.5–1km/h (Zheng et al., 2021), even though other authors refer the possibility of reaching 5km/h for on-the-go imaging (Kicherer et al., 2015; Gutiérrez et al., 2017).

### Infrared Thermography

Infrared thermography also shows potential for phenotyping in both controlled and field conditions, namely to assess drought stress (Jones et al., 2002; Costa et al., 2013; Diago et al., 2016). Stomatal conductance to water vapor correlates with the plant’s water status and regulates evaporative cooling, making plant temperature increase when stomata are closed. According to these principles there have been attempts to use thermography instead of the time-consuming leaf gas exchange measurements to assess plant water status and transpiration (Jones et al., 2002; Möller et al., 2007; Briglia et al., 2019). However, factors of environmental variability, such as wind speed, radiation, and air humidity could affect the robustness of thermal imaging data as compared to the actual plant status (Costa et al., 2013). The use of phenotyping vehicles following the concept of “mobile tunnel,” equipped with artificial broadband light sources, as is the case of the Phenoliner (Kicherer et al., 2017), may minimize those environmental disturbances. Another strategy to minimize environmental disturbances is the use of so-called thermal indexes. One of the most commonly applied, is the crop water stress index (CWSI; Clawson et al., 1989), based on the use of wet and dry reference surface temperatures. A high and stable correlation between CWSI and leaf conductance (g_L_) is found when CWSI is calculated using the temperature at the center of the canopy or its sunlight fraction (Clawson et al., 1989). A high positive correlation between g_L_ and stem water potential (ψ_stem_) during the season was also found (Irmak et al., 2000). Grapevine water status can be estimated through CWSI by using thermal imaging system and a RGB digital camera (Möller et al., 2007) in which the color image is used to select pixels with specific features, such as sunlit pixels, to create masks of soil and masks of shadowed leaves to enable the analysis of the temperature in the thermal images only in sunlit leaves. In turn, Matese et al. (2018) found a high correlation between CWSI obtained from proximal and remote thermal sensing and the physiological parameters net photosynthesis (Pn) and effective quantum yield of photosystem II (Fv′/Fm′) in the varieties Vermentino, Cabernet Sauvignon, and Cagnulari. Other reports emphasize the fact that canopy size and architecture, together with leaf orientation can result in different temperature readings for identical values of stomatal conductance (Grant et al., 2007; Grant et al., 2016). Furthermore, the use of wet and dry references, required to compute CWSI or other thermal indexes (e.g., stomatal conductance index – I_G_), may conflict with HTPP in field conditions, namely in air borne phenotyping. Therefore, alternative approaches, such as direct comparison between control irrigated and drought stressed plants, must be further developed.

An important and recent development in thermography, is the use of low cost equipment (e.g., thermal camera connected to a smartphone) to calculate water status indices, including CWSI and the stomatal conductance index (Petrie et al., 2019; Jouzier, 2020). Even though these instruments are less accurate, they are simpler and less expensive in monitoring plant stress responses and could also be used as pre-selection scanning to identify contrasting genotypes in terms of leaf/canopy temperature. However, when the expected temperature differences are small, such as in the case of studying intra-varietal variability, the effectiveness of this method is very limited.

Infrared sensors together with RGB sensors were also used in depth (3D) cameras. Recent technological advances that have been used for field phenotyping of grapevines have enabled the manufacture of consumer-grade depth cameras able to produce RGB information, infrared images, and 3D depth data (Milella et al., 2019). These systems might provide an alternative to the more expensive light detection and ranging (LiDAR) systems, in three-dimensional (3D) canopy reconstruction (Milella et al., 2019).

### Chlorophyll Fluorescence (Conventional and Imaging)

The emission of chlorophyll fluorescence (*CF*) is widely used as a contactless method to assess photochemical use of energy and its non-photochemical dissipation (NPQ). The intensity of *CF* is variable over time depending on the photosynthetic activity and has been used to estimate plant stress, maximum potential PSII efficiency (Fv/Fm), quantum yield, and electron transport rate (ETR). Chlorophyll fluorescence has been extensively used in the assessment of biotic and abiotic stress evaluation in grapevine (Su et al., 2015; Carvalho et al., 2016; Ju et al., 2018) and it has been introduced in HTPP (Marques da Silva, 2016). At leaf level, it is measured mostly with two classes of instruments: pulse amplitude modulating fluorometers and continuous excitation fluorometers. Chlorophyll fluorescence induction (CFIN) is widely used in stress physiology research related to photosynthesis as it provides several relevant information and it is both non-destructive and cheap (since it uses continuous excitation fluorometers, much cheaper than modulated fluorometers; Humplík et al., 2015). *Vitis* species present significant interspecific and intervarietal differences in the patterns of rapid fluorescence induction (Marques da Silva et al., 2020). However, conventional fluorometry (modulated or continuous) is a point measurement, where the signal is collected generally by an optical fiber that is in contact to or in close proximity to the leaf. This means that leaves have to be manually selected and processed, making automation impossible and thereby excluding these techniques from HTPP processes. On the contrary, chlorophyll fluorescence imaging (CFI) can collect whole-plant images and might be, therefore, included in HTPP platforms.

The use of CFI allows the study of spatial and temporal heterogeneities in fluorescence emission patterns at the level of cells, leaves, plants, or a whole field, and has potential use to identify stress tolerance and for genotype screening in breeding programs (Gorbe and Calatayud, 2012; Osório et al., 2014; Cen et al., 2017; McAusland et al., 2019; Sánchez-Moreiras et al., 2020). CFI is useful to asses stomatal patchiness and heterogeneity of photosynthetic activity (Omasa and Takayama, 2003), overcoming the problems of point measurements due to the high variability at leaf level (Ehlert and Hincha, 2008). Furthermore, imaging fluorimeters may allow the measurement of several samples (replicates) at the same time. However, assessment of fluorescence parameters that require sample dark adaptation (e.g., Fv/Fm) is not feasible in field phenotyping, but informative parameters not requiring dark adaptation (e.g., the photosynthetic ETR) can be measured, although the requirement of a low intensity modulated measuring pulse poses technical difficulties for remote measurements. Fluorescence imaging is under rapid technical development and new instruments are now available (Herritt et al., 2020). Nevertheless, the high cost and limited operational performance in field can hinder their use in large scale field phenotyping in grapevine in the near future.

Relevant information on stress conditions can also be obtained from the analysis of the spectral signature of chlorophyll fluorescence, which is collected after laser excitation in the laser induced fluorescence technique (Gameiro et al., 2016). This technique does not require sample dark adaptation or close proximity to the sample and therefore might be suitable for field HTPP (Marques da Silva, 2016; Marques da Silva and Utkin, 2018).

### Multispectral and Hyperspectral Imaging

Several important photosynthesis-related parameters can be investigated through the spectral composition of the light reflected by the plant, fruits, leaves, and canopy. The principle is that reflectance differences are related to chlorophyll, carotenoids, nitrogen, or water content (Walter et al., 2015), in particular, the reflectance analyzed in the visible, near-infrared, and short wavelength infrared spectrum (SWIR). The latter is used for the estimation of plant’s water status. The reflectance can be measured by spectrometers (Barradas et al., 2021), which provide point measurements (low/absent spatial resolution, very high spectral resolution), and by multispectral or hyperspectral cameras, which provide images (high spatial resolution, low/very low spectral resolution). A multitude of reflectance indexes have been published (for review, see Xue and Su, 2017), but most are suitable only for spectroscopic measurements, since they require the input of reflectance obtained at specific wavelengths, i.e., in a very narrow spectral band. However, as discussed above, point measurements are not suitable for HTPP and, therefore, multispectral or hyperspectral imaging is necessary. These measurements, initially used for remote sensing analysis of natural ecosystems, are also suitable for plant/crop phenotyping (Humplík et al., 2015), the main limitation, as for the canopy temperature analysis, is the spatial variability to which the plants are subjected during the measurement, and also the very high costs of the hyperspectral cameras (Table 1). SWIR measurements are at the basis of indices like the normalized difference vegetation index (NDVI), an estimator of the chlorophyll (chl) content, and the proportion of chl *a* in relation to chl *b*, and the photochemical reflectance index (PRI) which allows to estimate the photosynthetic efficiency by measuring the redox status of carotenoids (Humplík et al., 2015) that is part of the non-photochemical de-excitation pathway (Demmig-Adams et al., 2012), and in turn is correlated with photosynthetic light use efficiency (Sims and Gamon, 2002). A portable apparatus for NDVI ground-based measurement was tested in grapevine to estimate plant vigor by vine leaf area index (VLAI; Drissi et al., 2009), and the authors reported that the sensor is adequate to estimate plant vigor as VLAI and canopy gap, but only before the canopy growth saturates the response.

Two indices based on reflectance measurements, R690/R600 and R740/R800, where R690 and R740 are the chlorophyll fluorescence emission peaks and R600 and R800 are bands not affected by chlorophyll fluorescence, have been used to indirectly track changes in steady-state chlorophyll fluorescence due to heat and water stress (Dobrowski et al., 2005). Both indices had a strong positive curvilinear relation with steady state fluorescence (Fs).

More recently, Gutiérrez et al. (2018) showed the feasibility of a novel approach to classify leaves from several grapevine varieties grown in field conditions. The authors used on-the-go hyperspectral imaging at considerable speed (5km/h) and different machine learning algorithms.

Near infrared (NIR) hyperspectral imaging was also used to accurately predict anthocyanin content and evolution during development of Cabernet Sauvignon grapes from veraison to ripening (Chen et al., 2015). Also, NIR hyperspectral imaging was used to predict the quantification of total phenolic anthocyanins and flavanols in grapes of two red varieties, Syrah and Aragonez (syn Tempranillo; Nogales-Bueno et al., 2015). The results identified quantifiable differences between the two varieties regarding these parameters and, interestingly the authors observed a large range of distribution of values in each variety. Another study, performed in red and white varieties of table grapes, successfully used NIR hyperspectral imaging to predict sugar, total flavonoid, and total anthocyanin contents (Gabrielli et al., 2021). Sen et al. (2016) showed that the combination of visible and mid infrared (MIR, 4,000–650cm^−1^) ranges with methods of multivariate analysis improved the prediction of anthocyanin compounds and total phenols in wine as opposed to using NIR range alone. All these studies emphasize the importance of the robustness of the models adjusted. Furthermore, these traits are subject to high environmental variability, which can significantly change the rates of accumulation and degradation of sugars, flavonoids, and anthocyanins (Rienth et al., 2021). Also, intracluster berry heterogeneity can also be a main bias for individual berry phenotyping (Rienth et al., 2021). However, berry composition parameters have been used in grapevine selection (Table 2) with a high degree of success, and the effects of the environment can be overcome with an appropriate experimental set up and with sampling in several seasons (Gonçalves et al., 2016). Therefore, it may be possible to apply NIR hyperspectral imaging to clonal phenotyping to obtain data on berry composition for selection.

**Table 2:** Non-exhaustive list of phenotypic traits used in studies focused on agronomic, morphological, and eco-physiological characterization of grapevine genotypes.

Plant Material	Traits quantified at the following levels	
	Morphological and biophysical	Metabolic
Leaves	Individual area Color Shape Vein density Thricome density Cuticle thickness Mesophyll thickness Chlorophyll content Relative water content Temperature Water status Intrinsic water use efficiency	Carbohydrate ABA content
Cluster	Weight Color Number of berries Length and width Compactness Temperature Volume Projected area	
Trunk/shoots	Diameter Volume Shoot length	Carbohydrates
Roots^*^	Size Density Inclination	Carbohydrates
Canopy/Whole-plant	Yield Biomass/vigor Shoot length Exposed leaf area Number of leaf layers 3D leaf area Projected leaf rea Leaf area index Light penetration	
Berries/Must		Acidity pH °Brix Anthocyanins Phenols Aroma precursors

* Only for rootstock characterization and selection; traits in bold are already used for selection.

### Light Detection and Ranging

Light detection and ranging (LiDAR) is an active sensing technology that emits short-wavelength lasers, that can be visible, ultraviolet, or near- infrared light, to measure the distance from the sensor to the target according to laser speed and flight time recorded by a timer (Lin, 2015). These measurements are then translated into a 3D structure, built on the angle of the emitting laser collected by an angle encoder. LiDAR has some advantages, such as high throughput, high spatial resolution, high reproducibility, and the characteristics that make it suitable for field measurements, independency from light conditions, and the ability of the short-wavelength laser to penetrate the vegetation canopy (Jin et al., 2021). LiDAR sensors were first used in viticulture in 1998 to estimate several viticultural indices characterizing foliage distribution as well as attributes of the light microclimate in the canopy (Mabrouk and Sinoquet, 1998). The values obtained correlated well with those obtained by traditional methods and the authors were able to calculate bunch exposure and relate it with grape composition, namely sugar content, anthocyanins, and phenolics (Mabrouk and Sinoquet, 1998). This represented a major breakthrough in the estimation of key viticultural traits in an indirect, fast, reproducible, and non-destructive approach. The geometry of plant canopies can also be calculated using LiDAR, during the winter dormancy period, to calculate pruning weight, a previously laborious but extremely informative parameter to calculate plant vigor (Tagarakis et al., 2013, 2018). LAI was also successfully estimated with a laser sensor (Arnó et al., 2013), the authors obtained good correlations between LAI and canopy volume, as well as between LAI and tree area index. Nowadays, automated mobile platforms that move along rows scanning the vines are available. They are able to identify different managing systems and to calculate pruning weight, trunk, and cordon volume (Siebers et al., 2018). Water deficit can also be indirectly calculated through the measurement of plant leaf area, as it correlates well with the apparent soil electrical conductivity (EC_a_), giving an indication of the plant’s water needs (Tsoulias et al., 2019).

### Evaluating Success of Phenotyping for Plant Breeding and Selection

A well planned phenotyping procedure is a critical task in plant breeding because it is the starting point for any efficient selection of plants, as illustrated for grapevine in Figure 1. When working with quantitative traits (the most frequent and economically important ones, such as yield, tolerance, or quality), it is necessary to understand the meaning of the obtained phenotypic value. This requires the quantification of the part of the measured trait that is due to the genotypic causes.

In classical models of quantitative genetics (i.e., balanced data with no random effects other than those associated with genotypes and error, and diagonal variance-covariance matrices), the proportion of total variance (phenotypic variance) that is genetic is called broad sense heritability (Falconer and Mackay, 1996). At the level of the mean of the genotypes, the classical concept of broad-sense heritability ($H^{2}$) is defined as


$$H^{2}=\frac{\sigma_{g}^{2}}{\sigma_{g}^{2}+\frac{\sigma_{e}^{2}}{r}}$$

where $\;\sigma_{g}^{2}$, $\sigma_{e}^{2}$, and r are the genotypic variance, error variance, and number of replicates, respectively. The broad sense heritability is an important indicator of the quality of the experimental design of the trials for the evaluation of a target quantitative trait and, consequently, of the success of genetic selection. Due to its importance in the context of plant breeding of quantitative traits, several studies addressed the problem of defining the establishment of a generalized measure applicable to more complex models (Cullis et al., 2006; Oakey et al., 2006; Piepho and Möhring, 2007; Welham et al., 2010), including in the context of grapevine selection (Gonçalves et al., 2013). To summarize all these approaches, an approximate generalized measure of broad-sense heritability can be presented as


$$H^{2}=1-\frac{\bar{PEV}}{\sigma_{g}^{2}},$$

where $\bar{PEV}\;$is the average of the predicted error variance of genotypic effects and $\sigma_{g}^{2}$ is the genotypic variance.

Another key concept is the prediction of genetic gain (R) for the several traits evaluated. In the context of ancient grapevine varieties and under the classical models, it is defined as


$$R=S\times H^{2}$$

where S is the differential of selection, that is, the difference between the selected group of genotypes and the mean of the population and $H^{2}$ is the broad-sense heritability (Falconer and Mackay, 1996). Similarly, the genetic gain of selection is the mean of the Empirical Best Linear Unbiased Predictors (EBLUPs) of the genotypic effects of the top-ranked selected genotypes. This last definition is also applicable for more complex models. A selection based on EBLUPs of the genotypic effects of the best model would be more efficient and lead to higher genetic gains.

To quantify and obtain high values of heritability and high predicted genetic gains, that is, to achieve precision and accuracy in the evaluation of quantitative traits, agronomic experiments demand a well-planned phenotyping, which involves the establishment of field trials with efficient experimental designs (with repetition, randomization, and efficient control of spatial variation) and correspondent appropriate models for data analysis (mixed models).

Agronomic experiments are usually large, expensive, and take many years to accomplish. Additionally, they are typically subject to high background variability due to soil fertility and availability of water trends in the field (spatial variation) and cultural techniques, and other environmental deviations. This variability must be controlled through the type of experimental design. Typically the effective control of background variability is made by blocking or by using covariates together with sufficient replication of genotypes (Piepho and Edmondson, 2018). The experimental designs used in agriculture to reach these objectives have a long history and are routinely used in agronomic experiments, such as randomized complete block designs, latin squares, split-plot designs, and the family of incomplete block designs (Giesbrecht and Gumpertz, 2004). Nowadays, precision agriculture tools (e.g., soil water sensors, EC, and NDVI maps) can also be used to optimize the establishment of the experimental design in the field. These tools can help find homogenous patterns of soil composition and water availability that enable the definition of incomplete and complete blocks.

In grapevine, field trials for polyclonal selection comprise a representative sample of the intravarietal variability of the variety under study (Martins and Gonçalves, 2015; OIV, 2019). The experimental designs useful for screening a large number of genotypes and to provide reliable guidance to select the best genotypes are described in Gonçalves et al. (2010), and the most efficient are alpha designs and resolvable row-column designs.

The application and testing of HTPP methodologies in field trials with adequate experimental designs and the need to quantify the quality of the measurements obtained are constant concerns in the plant breeding context. For example, Tattaris et al. (2016) used spring wheat lines trials, established under an alpha-lattice design, with either two or three replications, to test HTPP monitoring of plant physiological traits (canopy temperature and a vegetation index). Tattaris et al. (2016) compared three remote sensing approaches using a low flying unmanned aerial vehicle, with that of proximal sensing, and satellite-based imagery to determine the most viable approaches for large scale crop genetic improvement. The results obtained supported the use of those techniques for HTPP for both precision and efficiency. In turn, Singh et al. (2019) demonstrated the considerable power of unmanned aerial systems or drone-based phenotyping as a HTPP alternative to visual assessments for the complex phenological trait of lodging, which significantly impacts yield and quality in many crops including wheat. They tested and validated quantitative assessment of lodging on 2,640 wheat breeding plots over the course of 2years using differential digital elevation models. A total of 590 and 595 unique wheat entries along with the check varieties were planted in alpha-lattice field design during seasons 2016 and 2017, respectively. The broad-sense heritability of visual and digital lodging measures ranged between 0.50 and 0.59. Andrade-Sanchez et al. (2014), proved that a tractor-based phenotyping system was capable of reliably acquiring and recording data for canopy temperature, height and reflectance on experimental plots of cotton plants throughout the growing season in the field. To prove that, they evaluated field trials with 25 Pima cotton cultivars arranged as a lattice design with four replications in a total of 200 plots. Measurements of canopy height, NDVI, and temperature all showed large differences among cultivars and expected interactions of cultivars with water regime and time of day. Broad-sense heritability ranged from 0.86 to 0.96 for canopy height, from 0.28 to 0.90 for the NDVI, and from 0.01 to 0.90 for temperature. Also in the context of high throughput phenotyping, Junker et al. (2015) highlighted some experimental procedures to optimize the quantitative evaluation of crop plant performance. In grapevine, Carvalho et al. (2020) evaluated abiotic stress tolerance, measured by the surface leaf temperature (SLT) of clones under environmental conditions of drought and extreme heat for 3years. SLT sets the boundary condition for the latent and sensible heat transport through vegetation, soil, and atmosphere, depending on the availability of moisture at the interface soil- atmosphere (Fuchs, 1990), giving an estimate of the response of a leaf to the environmental parameters affecting it at any time (air temperature, relative humidity, solar radiation, leaf resistance, and boundary layer resistance; Udompetaikul et al., 2011). By utilizing simple measurement devices and an experimental set up that enables the separation between environmental influence and the physiological response, it is possible to study the relationship between these parameters. A plant is able to keep a SLT lower than ambient temperature by controlling stomatal aperture and thus leaf gas exchange through stomata. The capacity to control stomata opening and thus CO_2_ intake for photosynthesis regardless of high air temperature gives the clones that hold it an advantage to face heat stress without loss of yield and quality of the grapes produced. The application of the methodology was done in a field trial with 255 different clones established according to a resolvable incomplete block experimental design with five complete blocks: each complete block comprised the effect of the complete block and the effect of the day; each column within each complete block, with approximately 13 plots, constituted an incomplete block, which comprised the effect of the time of day. With this type of experimental design, it was possible to prove the existence of significant genetic variability within the variety for the trait SLT and the values of generalized broad sense heritability ranged between 0.44 and 0.54, corresponding to a quantifiable genetic component difference of 3°C between the coolest and warmest of the 255 genotypes measured in three consecutive seasons.

In short, in the context of plant breeding, to perform fast, massive, or HTPP, the establishment of field trials with adequate experimental designs and the estimation of several genetic and statistical parameters, that provide information about the meaning and the quality of the data obtained, is mandatory.

### Traits to Use in Phenotyping for Selection

A more sustainable viticulture must involve the use of locally adapted varieties and selected material of those varieties. Phenotyping must enable a reliable identification of genotypes with the desired traits, whether yield, specific berry composition, or tolerance to stress and should contribute to estimate their genotypic diversity.

So far, grapevine selection within ancient varieties relies on the exhaustive gathering of specific data from all the genotypes in an experimental field (with biological replicates it generally reaches more than 3,000 plants; Martins and Gonçalves, 2015). Any possibility of automation without loss of reproducibility or precise quantification should be very welcome. Moreover, the data gathered are so dependent of the effects of the environment that only an efficient experimental design allows to control those effects, and most importantly, to quantify the contribution of the genetic component, the so called broad sense heritability.

Therefore, an effective selection of grapevine genotypes takes several years, requiring much labor and costs. The need to evaluate hundreds of genotypes in several repetitions occupies between 1.0 and 2.0ha and an efficient control of the field installation cannot allow the use of ready-made grafted plants. With all these constraints, such trials are only viable for economically prized varieties.

With respect to the data gathering itself, currently, only yield and berry composition have been exhaustively tested and quantified in selection trials (Gonçalves et al., 2016; Table 2). Yield is quantified by handpicking and weighing the production of each plant in the trial. However, the most time- and labor-consuming task of selection is berry composition analysis, which requires collection of individual berry samples from all grapevines and making the quantifications of pH, total acidity, and soluble solids and, in red varieties, anthocyanins, and phenols, in the lab, following standardized and well-established protocols.

The use of traits such as leaf temperature or the simpler RGB offer still limitations in assessing properly yield but mostly berry quality traits. More testing to find robust correlations between Tleaf or leaf color or canopy size and yield and quality are needed therefore, to make them used in selection for berry composition and final yield per plant.

## Conclusion and Prospects

Screening and characterization of inter- and intravarietal variability of autochthonous grapevine varieties has a crucial importance not only for the Portuguese but also for the global wine industry. The future competitiveness and higher sustainability of the sector should be largely based on the use of well adapted plant material (rootstocks and varieties). Drought and heat stress are major driving forces for grapevine selection and breeding as means to identify the most resistant and better adapted grapevine varieties/genotypes. In fact, one of the medium/long term strategies to respond to climate change adversities and the problems of increased stress is based on the selection and use of superior genotypes.

A major future challenge for grapevine field phenotyping is to exhaustively evaluate relevant traits for selection purposes, such as tolerance to abiotic stress. Moreover, the available imaging technologies (e.g., RGB, thermal, and multispectral) need to be adapted and optimized to large field trials (Table 1) to provide reliable quantitative data for a robust, reproducible, and comparable analysis at different levels (leaf, canopy, berry, and cluster). This task can be particularly challenging when dealing with intra-varietal characterization and clonal selection, attending to the potentially smaller differences between genotypes for some traits, namely those related to tolerance to abiotic stress. Also, the possibility for automation of data gathering for traits already under analysis, such as berry composition, would expedite measurements of large experiments.

Proximal and remote sensing technologies have undergone great developments in recent years and have become more accurate, cheaper and, in some cases, more user-friendly. The attention of the scientific and industry communities toward these technologies is very high due to their potential for field analysis and subsequent management of variability in field conditions. In viticulture, they are chiefly applied in the agronomic management of the vineyard as part of the so called “precision viticulture,” but some proximal and remote sensing technologies have potential for phenotyping and selection. For this purpose, it will be necessary to deepen and clarify the link between the indirect digital measurements obtained by sensors and the morphological, eco-physiological, and metabolic parameters under examination, which sometimes is still doubtful. The following step would be to develop specific and standardized protocols to apply these sensing technologies to grapevine phenotyping in field conditions, mainly focused on leaf, berry, or canopy/plant traits that are closely related to physiologically complex phenomena, such as that of tolerance to abiotic stress.

The advance in imaging technologies, robotics and computing will enable to establish and perform new assays for genotype characterization and selection that can be carried out under field conditions. This can also provide more tools to study grapevine development and behavior under climate change conditions.

## Author Contributions

JC: idea, writing, editing, reviewing and funding. LC and EG: writing, editing, and reviewing. JMS: writing, reviewing, and funding. All authors contributed to the article and approved the submitted version.

## Funding

This work was supported by the Fundação para a Ciência e Tecnologia (FCT) research unit BioISI (UID/MULTI/04046/2019) and the FCT funded R&D project INTERPHENO (PTDC/ASP-PLA/28726/2017). LC acknowledges the funding by FCT through DL57/2016/CP1382/CT0024. Linking Landscape, Environment, Agriculture, and Food (LEAF) research center has been funded by national funds through FCT – Fundação para a Ciência e a Tecnologia, I.P., in the scope of the projects UID/AGR/04129/2013; UID/AGR/04129/2019, and presently UIDB/ and UIDP/04129/2020.

## Conflict of Interest

The authors declare that the research was conducted in the absence of any commercial or financial relationships that could be construed as a potential conflict of interest.

## Publisher’s Note

All claims expressed in this article are solely those of the authors and do not necessarily represent those of their affiliated organizations, or those of the publisher, the editors and the reviewers. Any product that may be evaluated in this article, or claim that may be made by its manufacturer, is not guaranteed or endorsed by the publisher.

## Footnotes

^1^ https://www.plant-phenotyping.org/

^2^ http://interpheno.rd.ciencias.ulisboa.pt/

^3^ https://emphasis.plant-phenotyping.eu/
